# Fertility Desires among Men and Women Living with HIV/AIDS in Nairobi Slums: A Mixed Methods Study

**DOI:** 10.1371/journal.pone.0106292

**Published:** 2014-08-29

**Authors:** Eliud Wekesa, Ernestina Coast

**Affiliations:** 1 Population Council, Nairobi, Kenya; 2 London School of Economics, London, United Kingdom; International AIDS Vaccine Initiative, United States of America

## Abstract

**Objectives:**

Fertility desires require new understanding in a context of expanding access to antiretroviral therapy for people living with HIV/AIDS in Sub-Saharan Africa. This paper studies the fertility desires and their rationales, of slum-dwelling Kenyan men and women living with HIV/AIDS who know their serostatus, but have different antiretroviral therapy treatment statuses. It addresses two research questions: How do people living with HIV/AIDS consider their future fertility? What factors contribute to an explanation of fertility desires among people living with HIV/AIDS.

**Methods:**

A mixed methods study (survey [n = 513] and in-depth interviews [n = 41]) with adults living with HIV/AIDS living in Nairobi slums was conducted in 2010. Regression analyses assess independent relationships between fertility desires and socio-demographic factors. Analyses of in-depth interviews are used to interpret the statistical analyses of fertility desires.

**Results:**

Our analyses show that fertility desires are complex and ambivalent, reflecting tensions between familial and societal pressures to have children versus pressures for HIV (re-)infection prevention. More than a third (34%) of men and women living with HIV expressed future fertility desires; however, this is significantly lower than in the general population. Factors independently associated with desiring a child among people living with HIV/AIDS were age, sex, number of surviving children, social support and household wealth of the respondent.

**Discussion:**

Increasing access to ART is changing the context of future childbearing for people living with HIV/AIDS. Prevailing values mean that, for many people living with HIV/AIDS, having children is seen as necessary for a “normal” and healthy adult life. However, the social rewards of childbearing conflict with moral imperatives of HIV prevention, presenting dilemmas about the “proper” reproductive behaviour of people living with HIV/AIDS. The health policy and service delivery implications of these findings are explored.

## Introduction

Sub-Saharan Africa (SSA) is the region with the highest numbers of people living with HIV/AIDS, and the highest adult (15–49 years) HIV prevalence [1]. Treatment coverage expansion of antiretroviral therapy (ART) has transformed HIV/AIDS into a manageable chronic illness for many [2]. In Kenya, it is estimated that seventy-three per cent of adults and children eligible for treatment have access to ART [2]. The changing nature of living with HIV on ART poses questions about the implications of this treatment, over and above increased well-being and reduced mortality and morbidity risks. The implications of ART for HIV-infected individual’s (or couple’s) future fertility is generating growing interest [3], [4]. Positive associations between ART and fertility desires have been found in South Africa [5]; Uganda [4]; Brazil [6] and India [7]. One study reported a significant negative association between ART use and fertility desires in Nigeria [8]. No association between ART use and fertility desires has been documented in Uganda [9] and South Africa [3].

Most people living with HIV/AIDS (PLWHA) in sub-Saharan Africa are in their prime child-bearing and rearing years, many are already parents, and live in a context where a high premium is placed on parenthood [10]. Many PLWHA continue to desire and have children after knowing their HIV status [4], [11]. Unlike the general population, however, people who know they are HIV infected have additional issues to consider, including potential health risks for (re)infections, vertical transmission of HIV and potential orphaning of existing and future children [12]. For some PLWHA, meeting family and social obligations concerning reproduction, may be more important than the risk of HIV transmission [10]. For others, however, their health status and risk of HIV transmission might dissuade them from wanting a/another child [13]. These issues present PLWHA with a dilemma of either risking vertical and/or horizontal transmission of HIV or else setting aside fertility desires [12]. Among PLWHA – as in the general population – the pleasure, happiness and fulfillment associated with parenthood, familial and societal pressure [10], the need to carry the family name and perpetuate the lineage [14] and the need for securing a marriage or relationship [15] – are some of the reasons for wanting children.

Fertility desires of PLWHA are complex and shaped by different, sometimes conflicting, sets of factors, some of which apply to the general population, while others apply specifically to PLWHA. Factors that apply to the general population include demographic (e.g.: age [5], gender [11] and socio-cultural (e.g.: norms and values about parenthood [10], [15]) influences. Factors that are more likely to be of importance to PLWHA include subjective health status [16], availability of ART and prevention of mother-to-child transmission (PMTCT) programmes [3], the availability of social support [17], and whether they have disclosed their status [15].

Nearly three quarters (72%) of urban residents in Sub-Saharan Africa live in slums [18] and up to 70% of Nairobi residents live in slums or slum-like areas [19]. Our data are based on evidence collected from slum- dwellers, an under-researched population, and reflects the need for evidence that better understands the contemporary lives of many African urban residents and rural-urban migrants. The study was conducted in two slum communities (Korogocho and Viwandani) in Nairobi City, Kenya. The housing structures in these slums are temporary, typically single rooms constructed from mud, iron sheets, cardboard boxes and polythene paper [20]. The two communities are characterised by overcrowding, insecurity, poor housing and sanitary conditions, lack of basic social amenities and infrastructure, extreme poverty, high unemployment levels, crime, low educational levels, limited access to preventative and curative health services and reliance on poor quality, usually informal and unregulated health services [19]. These conditions combine to produce poor health outcomes for slum residents compared to other populations in Kenya, including higher rates of mortality, morbidity, HIV prevalence, riskier sexual behaviours, unmet need for family planning and unintended pregnancies [19], [21]. Because these settlements are illegal, government authorities are reluctant to provide them with infrastructure and amenities. These settlements are beginning to attract attention from non-governmental organisations and community-based organisations, including faith-based organisations (FBOs) [22].

This paper analyses the fertility desires, and their rationales, of slum-dwelling men and women living with HIV/AIDS who know their serostatus, but have different ART treatment statuses. It addresses two research questions: How do men and women living with HIV/AIDS consider their future fertility? What factors contribute to an explanation of fertility desires among PLWHA?

## Materials and Methods

A mixed methods study involving survey and in-depth interviews with PLWHA (18 years and above) was conducted in 2010. The study adopted a sequential mixed methods design [23], which involved a phase of quantitative survey interviews (n = 513) followed by a qualitative phase on a subsample (n = 41) purposively drawn from the survey respondents. The quantitative sample size was determined on the basis of sample size calculations [24], calculated on the assumption that 50% of the sample would want to have a/another child(ren) in the future, with a sampling error of 5% and at 95% level of confidence. The eligibility criteria for inclusion in the survey was being HIV-positive, over 18 years and able and willing to provide informed consent to be included in the study.

Respondents were recruited from the Nairobi Urban Demographic and Health Surveillance System (NUDHSS) sites of Korogocho and Viwandani. In order to draw a representative sample of population, the study adopted targeted sampling, using sero- prevalence ratios in the study sites. A sero-prevalence study carried out separately in the study sites in 2006/7 showed that the following characteristics are associated with HIV Prevalence in the slums: sex, marital status, ethnicity, age and education [21]. These characteristics were proportionally represented in the survey sample (Table 1), which then provided a sampling frame for the subsequent in-depth interviews. In-depth interviewees were purposively selected on the basis of their responses to survey questions about sexual behaviour, fertility intentions, contraceptive use and antiretroviral therapy use in order to generate a sub-sample of typical cases. Of the 45 individuals approached for an in-depth interview, we interviewed 41 (three had moved and we were unable to trace them, one refused to be re-interviewed).

**Table 1:** Percentage distribution of socio-demographic characteristics of survey (n = 513) and in-depth interviewees (n = 41) living with HIV/AIDS in Nairobi slums.

CHARACTERISTIC	QUANTITATIVE		QUALITATIVE	
	N	%	N	%
**Slum residence**				
Korogocho	260	51	23	56
Viwandani	253	49	18	44
**Sex of respondent**				
Female	318	62	23	56
Male	195	38	18	44
**Age (years)**				
18–29	89	17	8	20
30–39	210	41	13	31
40 and above	214	42	20	49
**Marital status**				
Married/cohabiting	281	55	12	29
Divorced/Separated	91	18	9	22
Widowed	103	20	14	34
Never Married	38	7	6	15
**Ethnicity**				
Kikuyu	156	30	15	37
Luo	126	25	11	27
Luyia	104	20	7	17
Kamba	85	17	5	12
Other	42	8	3	7
**Living Children**				
0	27	5	2	5
1 or2	173	34	16	39
3 or 4	184	36	14	34
5 or more	129	25	9	22
**Treatment status**				
On ART (Ref)	268	52	18	44
Not on ART	245	48	23	56

The study employed community health workers (CHW) to help in the recruitment of respondents, and research assistants (RA) to conduct the quantitative and qualitative interviews under the supervision of EW. Eight (3 male and 5 female) RAs (four for each site) were recruited for the quantitative phase, of which two were retained for the qualitative phase. Four of the RAs had university degrees while the other four had diploma qualifications in counselling and were trained as HIV/AIDs counsellors, and one RA was a PLWHA. They all had experience of data collection in the study sites. Survey interviews were conducted in KiSwahili, and the in-depth interviews were recorded, transcribed verbatim, and translated into English. Field notes were made during the course of the in-depth interviews. Survey interviews lasted approximately a half an hour and in-depth interviews lasted approximately one and a half hours. A detailed description of the methodology is available elsewhere [25].

We obtained written consent from all respondents and all interviews were conducted in a setting of the respondent’s choice. Privacy in home settings in slums is difficult to achieve, and respondents were given the option of being interviewed in the offices of a local health organisation. A small grocery package was provided as compensation for each respondent, in line with local practices for social science research in these settings. Approval for the study was granted by the Kenyan Medical Research Institute and the London School of Economics. As part of our commitment to conducting ethical research, trained counsellors were available for a minority of respondents who were distressed as a result of narrating their experiences of living with HIV. Our analyses use pseudonyms for the presentation of data. Copies of the survey questionnaire and in-depth interview instruments are available [25].

### Instrument design and measurement: survey

Fertility desires in the survey were assessed using the question “Would you like to have a/another child or would you prefer not to have any (more) children?” Respondents who indicated that they wanted a/another child were asked about the total number of children they would like to have in future and their sex composition. They were also asked about how long they would like to wait before having a/another child. This battery of questions draws on the questions routinely asked in Demographic and Health Surveys. These measures are informed by the theory of planned behaviour (TPB), which posits that people hold preferences, desires, intentions, or plans regarding fertility that can be clearly articulated and that they act according to those beliefs [26]. However, it is well-established that people report inconsistent, contradictory and ambiguous fertility desires, intentions and motivations [27]. We, therefore, supplemented the survey data with qualitative in-depth interviews of a sub-sample drawn from the survey respondents.

The survey included questions on a range of factors including: demographic (gender, age, number of living children, marital status); health (ART use, subjective health status); Socio-economic (Education, household wealth); and, socio-cultural (cultural norms and values, disclosure of HIV status, stigma, social support). Most of these explanatory variables (e.g sex, ethnicity, marital status) were categorical. Some variables (e.g.: internalised stigma, psychological distress, social support) were assessed by item scales. The scores from the measurement scales were averaged into a composite score for each construct using Cronbach’s Alpha because, in addition to deriving a composite measure, it also assesses internal consistency or reliability of the measuring instrument. It is thus an indicator of how well the items form a single scale in measuring the same concept [28]. Household wealth was measured by wealth quintiles based on a wealth index generated from information on housing characteristics and a list of household assets using principal components analysis (PCA), which creates summary indices and derives PCA scores [29].

### Instrument design: in-depth interview

The guides, which were pilot tested, consisted of a series of open ended questions that were face to face interviewer-administered. Interviewers probed further after each response to elicit as many descriptions as possible. The in-depth interview guide for PLWHA included: Experiences around HIV testing, the impact of the diagnosis on their sexual and reproductive lives, fertility desires and family planning (including personal experiences and community attitudes). The questions were semi-structured with pre-determined topics to ensure full coverage of salient issues as well as flexibility for respondents to direct the conversation.

### Analyses

Quantitative analyses were conducted in Stata version 11 [30]. Descriptive and multivariate logistic regression statistics were used to assess independent relationships between fertility desires and a range of background variables. The regression coefficients of the logistic models were interpreted in terms of odds ratios, controlling for other explanatory variables. Variables that demonstrated a bivariate association with outcome variables at p<.05 were entered into a multivariate logistic regression to assess predictors of the outcome variables. Stepwise forward and backward model selection was used to develop a parsimonious model of significant predictors of fertility desires. Prior to running the final logistic regression models, explanatory variables were tested for interaction effects and multicollinearity. Qualitative interviews were recorded, transcribed verbatim, translated into English, coded and thematically analysed using NVivo 8 [31]. Qualitative data analysis followed four steps: 1) reading transcripts in order to develop a general sense of data; 2) coding the data; 3) generating themes; and, 4) making an interpretation and meaning of data and constructing a narrative [32]. The coding frame was developed, tested on a sample of transcripts by the authors and revised. The themes derived were data driven. Quantitative and qualitative data are integrated in our analysis and interpretation.

## Results

The two sets of data (survey and in-depth interviews) were used to address the same research questions during interpretation and reporting of the findings. Below, we present the quantitative and qualitative data in parallel in order to understand, qualify and elaborate each other. In our presentation of quotes from in-depth interviews, we include the question that elicited the response in order that the reader can situate the quote. Where we use quotes, all individual identifying (direct and indirect) information has been anonymised.

The majority (62%) of respondents were female and just over half (55%) of the sample were in a co-residential union (married or living with a spouse) (Table 1). About two thirds (65%) of the respondents had completed primary level education and 28% had received some secondary level education. The majority of the sample self-identified as member of one of four (Kikuyu, Luo, Luyia and Kamba) ethnic groups in Kenya. The majority (71%) of the respondents were between 30–49 years (mean age = 38). The proportions of those on ART and not were almost evenly split. However, women included in our sample were significantly more likely than men to be on ART. The mean duration of being on ART was also significantly longer for women than men, reflecting women’s likelihood of earlier testing (often linked to antenatal care), relative to men. Over four-fifths (83%) of respondents had known their HIV status for less than 5 years (mean = 3.2 years).

### Describing fertility desires

Two thirds (66%) of PLWHA do not want to have any/more children. However, among those that did want future child/ren, the levels varied substantially by sex, with 45% of men and 26% of women reporting that they had future fertility desires. Over one fifth (21%) of women and men with HIV/AIDS would like to wait two years or more for their (next) child, and 12% would like to have a/another child within two years.

Such quantitative description based on survey responses may imply that respondents were able to clearly state and articulate their fertility desires. However, this does not necessarily hold true. When we consider our qualitative data we see that respondents’ fertility desires could sometimes be ambiguous and over the course of a single interview could change and become contradictory, both between the survey and the in-depth interview and within the in-depth interview. Fertility desires are revealed as ambivalent linked to uncertainties about the future. A widow, who is expecting to remarry soon, expresses these feelings about her future child bearing, although she had previously indicated in the survey that she wanted another child:

INTE: You said that you want another child, right?

KC04: Well, I might. It is normal to have children, but I can’t have more than one child with him so that they are just four in total. But my mind does not want to give birth at all, because of my status. You know we were told [during counselling] that if you know your status you should not give birth frequently and shedding blood, as this may weaken you. So I don’t know.

(Widow, aged 27, mother of 3 living children)

Similarly, a male respondent presents apparently contradictory information about future children he might father. In the survey he replied that he would like to have another child, and he reiterates this at the start of the in-depth interview. But when asked why he would like to have another child, his response changes:

INTE: last time [survey] you said that you would you like to have another child? KC09: Yes, when I get another wife, I will get a baby… it is the right thing to do

INTE: Why would you like to have another baby?

KC09: If you get another child who is HIV-positive why should you have another one? You have brought a burden in your life; your household will be ailing all the time.

INTE: So you do not wish to have a child or…?

KC09: I don’t want [any more children]…I have borne many children…some have died and others are alive. One was killed by the M*ungiki* (a gang) and I was left with one here, others are married.

(Widower aged 55, father of 3 living children)

Such superficially contradictory responses are not simply about the mode of data collection (survey versus in-depth interview); they reflect uncertainty about the future.

INTE: The last time you said you did not want to get any children? VB01: I said I did not want a child at all.

INTE: Why did you say you did not want more children?

VB01: I don’t want a child right now, later I may get a child, but not for now.

(Mother of 1, 34 years, unmarried)

In many respects the more detailed qualitative responses reveal an attitude of “wait and see”. Men and women living with HIV want to wait and see how their health, material and marital conditions evolve, thus making explicit fertility desires difficult to articulate unambiguously.

### Factors influencing fertility desires

Fertility desires reported in the survey were independently significantly associated with sex, age, number of living children, social support and household wealth (Table 2).

**Table 2:** Multivariate parsimonious model of predictors of fertility desire among men (18–59) and women of reproductive age (18–49 years) living with HIV/AIDS in Nairobi slums (n = 463).

CHARACTERISTIC	FERTILITY DESIRE TO HAVE A (NOTHER) CHILD		RISK ESTIMATES	
	YES (N = 157)	NO (N = 306)	ADJUSTED OR (95% CI)	VALUE
**Sex of respondent**				
Female (Ref)	71 (26%)	199 (74%)	1.00	
Male	86 (45%)	107 (55%)	4.17 (2.46–7.07)	<0.001
**Age**				
40 years and above (Ref)	45 (25%)	137 (75%)	1.00	
18–29 years	41 (48%)	45 (52%)	2.50 (1.18–5.28)	0.017
30–39 years	71 (36%)	124 (64%)	1.57 (0.89–2.78)	0.119
**Living children**				
5 or more (Ref)	14 (13%)	96 (87%)	1.00	
0 (No child)	19 (79%)	5 (21%)	29.07 (8.51–99.33)	<0.001
1 or 2	88 (55%)	83 (45%)	8.99 (4.29–18.83)	<0.001
3 or 4	36 (21%)	132 (79%)	2.03 (0.97–4.24)	0.059
**Wealth quintile**				
Poorest (Ref)	23 (26%)	67 (74%)	1.00	
Second	32 (33%)	64 (67%)	1.54 (0.74–3.22)	0.250
Third	44 (40%)	67 (60%)	2.15 (1.06–4.37)	0.033
Fourth	26 (35%)	65 (48%)	2.19 (1.01–4.76)	0.048
Richest	32 (34%)	60 (66%)	1.68 (0.80–3.51)	0.172
**Treatment status**				
On ART (Ref)	85 (36%)	152 (64%)	1.00	
Not on ART	72 (32%)	152 (68%)	0.75 (0.47–1.19)	0.222
**Social support**	–	–	1.59 (1.08–2.34)	1.019

The odds of desiring a/another child for men were 4.17 times higher those of women, adjusted for other factors (OR 4.17; 95% CI 2.46–7.07; p = <0.0001). In sub-Saharan Africa, the premium for more children can be higher for men than women, particularly in patrilineal lineage systems, which predominate in most Kenyan ethnic groups, in which children belong to the paternal clan:

INTE: Last time you said that you wanted to have more children, why is it important to you?

VC05: It is important because that is taking the community or family forward. It is for continuity of your lineage and clan.

INTE: Can you explain further on this concept?

VC05: We always refer to children as the bearers of the family name. So if you don’t have a child no one will be named after you. Your child will name after you whenever he/she gets a kid. Again, if you don’t have children you won’t have someone to take care of you in the old age.

(Divorced father of 4, 52 years)

In the in-depth interviews some respondents mentioned strong societal values and pressure to have children as a factor that may compel them to either start or continue childbearing:

INTE: What is the importance of children in your tribe?

VC11: In my community if you don’t have a child you are not taken as a human being. So it is a must to have children. A child gives you a name and respect within the community. The child also offers support to the parents in their older age. And you also know that if you have no children then other people will come and take over your property when you die.

(Married Polygamous man aged 46, father of 11)

INTE: What is the importance of children in your tribe?

KB08: For women, it is very important to have a child. It gives you security…even if your *Mzee* (husband) dies you will not be chased away. If you don’t have a child your husband can even start having extra-marital affairs or marry another one.

(Married, mother of 2, aged 25)

Age and parity of the respondents were independently inversely related to fertility desires. Those aged 18–29 were as twice as likely as those aged 40 and above to want future fertility (OR 2.50), while the odds of fertility desires of those with one child were more than 29 times higher than those with 5 or more children (OR 29.07 95% CI 8.51–99.33; p = <0.0001)). PLWHA, in keeping with the general population, continue to want (more) children until they achieve their desired family size. Relatively young PLWHA may not have started their childbearing or may not yet have achieved their desired fertility levels. Childbearing for some women can constitute a validation of marriage:

INTE: You said that you would want more children in the future. Why is that so?

KB10: I am still very young and I have only one child. If I just stay with this one like that then people might start wondering what is wrong with me, especially my mother. Besides, I have a girl child and in my community they value boys so much. This will bring out some marital problems in our house in the future. So I would want to give my husband a son

(Married mother of 1, 25 years)

Social support (measured by composite scores) was positively associated with fertility desires (OR 1.52; 95% CI 1.08–2.34; p = <0. 0.019). These results suggest that social support helps people cope with stigma and negative reactions from society in general and fears against having children in particular. It might also be that the existence of social support makes it easier to have children (e.g.: emotional support, child-care and advice). Those who disclosed their sero-status to their spouses might be more likely to have discussed fertility intentions together:

INTE: Have you discussed the issue of children with your wife?

KB06: We have discussed that issue with my wife several times; we even talked about it immediately we were diagnosed and continue doing so even now. So we agreed not to use a condom at some time so that my wife could conceive. We had sex without protection a number of times, without success, but somehow on one occasion she conceived. It was a surprise because we had sort of given up. She was sickly and when she went to hospital she was told that she was pregnant. She did not believe it either. It was great!

(Married father of 2, aged 36)

Some level of household wealth was associated with desiring fertility. Those in the 3^rd^ and 4^th^ wealth quintiles were more than twice likely to state a desire to have a/nother child when compared to those in the poorest quintile (OR 2.15 and 2.19) respectively. Poor respondents reported difficulties in raising children in the in-depth interviews:

INTE: Please tell me why you do not want any more children?

KA04: It all has to do with … the income that I get. Taking care of children is a heavy task in this *kijiji* (slum). Even the two that I have I am struggling to provide for, so it would be senseless adding others

(Never married woman, aged 40)

Expanding availability of ART and prevention of mother-to-child transmission (PMTCT) offer PLWHA the possibility of realising their fertility goals and some respondents mentioned it an important factor motivating their fertility desires:

INTE: Explain why you said giving birth to healthy children is possible for you?

KC04: Because there are drugs that will prevent the child from getting HIV, but that does not mean that you go there and give birth anyhow. The doctor can advise you on how to do it [birthing] without the baby getting the virus…And it depends on the individual. Maybe somebody got it [HIV] when she was 18, 20 or 24 years and therefore wants to get married and have a child. So you cannot deny them the right to do that. Many people [PLWHA] are trying it out with the hope that it works for them.

(Widowed mother of 3, aged 27)

However, worries about the possibility of vertical and horizontal transmission abound, even when these interventions are available. The perceived detrimental effects of pregnancy and childbirth on their health and immune status were often expressed by women:

KC03: If you get pregnant and your CD4 count is low… that is dangerous. If you give birth you willreally be affected. You risk your life and can die in the process and leave the child in problems.

(Married mother of 2, aged 26)

Childbearing intentions of PLWHA were, to some extent, reflective of a quest to regain some normalcy for social approval and moral validation. For some, having children was a way of showing normalcy, healthfulness and of being socially conformist:

KC05: Most people used to say that HIV positive people are a finished lot. But nowadays they just get shocked when they see PLWHA breastfeeding children. Having a child is very important and normal. You know now I gave birth when I didn’t have the virus. Now (with PMCT) why can’t I give birth while I am positive while I know that that kid will not have the virus, and now leave that HIV negative offspring behind?

(Married mother of 1, aged 31)

For others, the quest for moral validation may work against fertility desires because unprotected sex may transmit HIV to children and sexual partners, acts that were presented by some respondents as immoral:

KA17: I always hear that there are drugs that people are given to prevent the baby from getting infected with the virus. But then don’t you think that I will infect the person that I am going to sleep with (in order to conceive], with the HIV virus? I cannot give somebody the virus knowingly like that just for him to impregnate me and then go and take drugs that will prevent the child from getting the virus. What about that man? … God might bless me and I give birth to another child who is HIV negative and then I die shortly after that, and leave them to suffer..I don’t see the need of mistreating my children.

(Widowed mother of 2, aged 30)

Related to concerns about HIV transmission and deterioration of health, several PLWHA expressed the fear of dying prematurely and spoke of uncertainty around the length of their lives. What arose during our discussions about fertility desires was that some PLWHA considered that other PLWHA who have children after knowing their HIV status exhibited a lack of responsibility towards themselves, their future child or their existing family. For some people, this perceived lack of responsibility was considerably greater than the potential risk of vertical transmission of 1–2% in a PMTCT programme [33].

## Discussion

This study provides mixed methods evidence and insights into the fertility desires of PLWHA in Nairobi slums in the era of ART. This contribution is important, both in the context of the sexual and reproductive health service needs of PLWHA, as well as expanding ART access. The findings show that 34% of PLWHA (38% among currently married) desire future fertility, which is lower than the general population in Kenya (50%) [34] and Nairobi slums (57%) [35]. It should be pointed out that fertility desires among PLWHA were lower than in the general population, partly due to the fact that this was an older cohort (mean age 38) in comparison to the women and men of reproductive age in the general population.

Our findings confirm other findings (e.g [5], [11]) that infection with HIV depresses, but does not eliminate, desire for future fertility. A study from South Africa found that 29% of PLWHA wanted to have children in future [5]. In two separate Ugandan studies, 35% and 29% of PLWHA reported that they wanted to have more children respectively [9], [36]. In Nigeria, a substantially higher proportion (64%) of PLWHA expressed a desire to have more children [37].

Our findings corroborate other studies’ observation that fertility desires among PLWHA in SSA are characterised by complexity and influenced by a confluence of socio-cultural, demographic, socio-economic and health-related factors. The factors independently associated with desiring a child in the future among PLWHA in this study have similarly been documented elsewhere in SSA settings. Younger age was significantly positively associated with desire for more children among PLWHA in South Africa [5], Uganda [36] and Nigeria [8]. Gender has also been reported to influence fertility desires, with men being more likely than women to desire children in Uganda [38], Malawi [39] and South Africa [11]. However, one study in Nigeria [37] did not find any association between gender and fertility desires among PLWHA. Fertility desire has been reported to be negatively associated with number of living children among PLWHA in South Africa [3], [5], Uganda [38], Nigeria [37] and Malawi [39]. Social support was found to be associated with fertility desire in a Ugandan study [15]. The cultural value of children in the identity and social status of HIV-infected men and women has been reported in studies in South Africa [10] and Uganda [15].

Conversely, use of ART was not associated with fertility desire in our study, which confirms findings from Uganda [9], Nigeria [37] and South Africa [3]. Contradictory findings – a positive association between ART and fertility desires – have been noted in South Africa [5], [11]. The lack of a consistent association between ART use and fertility desires has been attributed to the fact that with rapidly increasing access to ART, those not yet enrolled are confident enough to enrol once they become eligible, thereby minimising the differences between the groups [3].

ART reduces both vertical and horizontal HIV transmission risks associated with pregnancy and childbearing. Observational studies [40], [41] and a clinical trial [42] among discordant couples have shown that earlier antiretroviral therapy (ART) initiation substantially reduces the risk of HIV transmission within sero-discordant couples. ART decreases infectivity among its users by reducing their viral loads, with virtually no transmission by PLWHA with undetectable viral loads [43]. This evidence has culminated into the phenomena of Treatment as Prevention (TasP), with WHO recommending early initiation of ART to infected partners in sero-discordant couples to reduce risk of HIV transmission to uninfected partners [44].

Our study was done before the advent of TasP and so its influence on fertility desires was not assessed. However, it is plausible to hypothesise that Tasp has a positive influence on fertility desires of women and men living with HIV/AIDS in the context of expanding ART coverage in Kenya. A study in East Africa observed a positive relationship between fertility desires and early ART initiation, and HIV sero-discordant couples desiring children were nearly twice as interested in early ART for HIV prevention compared to HIV sero-discordant couples not desiring (more) children [45].

Parenthood is an important facet of PLWHA’s social status and social approval as well [10], [14]. Infection with HIV is not only disruptive and a threat to individual life, but also a threat to parenthood and personal integrity. Having children should be seen as a quest by PLWHA to repair their “spoilt” identity and appear normal and health for social approval and mitigation of stigma [14]. However, childbearing by PLWHA carries with it an “immoral” aspect of HIV transmission. Therefore, the inherent social rewards of childbearing and its inherent risk of transmitting the virus are complex issues in reproductive decisions among PLWHA. Consequently, as the data shows, PLWHA face a dilemma about what constitutes “proper” reproductive behaviour. Childbearing among PLWHA faces tension between social pressures and moral pressures. As such fertility desires are fraught with ambivalence, reflecting a conflict between social and moral pressures.

### Limitations

Five limitations of our study design and data collection should be acknowledged, together with their implications for our findings. First, information on fertility desires were based on self-reports using face to face interviews and so there is likely to have been social desirability bias. Men and women living with HIV/AIDS may underreport their fertility desires as result of perceived negative social approval from the community and health care providers. Second, the cross-sectional nature of our study precludes the determination of causality and the ability to understand changing fertility desires, which are likely to change with duration of HIV and ART. Thirdly, our study was done before the advent of TasP and so its influence on fertility desires was not assessed. However, it is plausible to hypothesis that Tasp has a positive influence on fertility desires of women and men living with HIV/AIDS in the context of expanding ART coverage in Kenya. Fourth, this was not a strictly probabilistic sample, but rather a sample of PLWHA who self-identified as being HIV positive living in the NUDHSS sites in Nairobi slums. Although we used targeted sampling, on the basis of previous work in order to have as representative sample as possible from which to draw valid conclusions, this limits the generalisability of the findings. Finally, our study focuses on individual level factors that influence fertility desires. Whilst some meso- and macro-level factors are likely to influence these individual-level factors (eg: residence in a slum community or ethnicity as it influences norms about lineage), they are not explicitly incorporated into our study design.

### Conclusions

Increasing access to ART and PMCT programmes is changing the landscape of fertility desires, allowing some PLWHA to desire and realise their reproductive goals. Prevailing pronatalist values and stigma against HIV/AIDS in most sub-Saharan Africa contexts means that, for many PLWHA, having children is seen as necessary for “normal” and healthy adult life. However, the social rewards of childbearing conflict with moral imperatives of HIV prevention, presenting a dilemma about “proper” reproductive behaviour of PLWHA. This means that the fertility desires of PLWHA are fraught with ambivalence and ambiguity, irrespective of their treatment status.

PLWHA in slums are under (sometimes opposing) pressure from different sources about whether to have (more) children: traditional expectations, cultural and religious values on procreation; economic constraints of an urban life; new family size values, and sexual and reproductive health service providers [36], [46]. Because peoples’ reproductive desires and preferences have to negotiate these multiple and sometimes incompatible socio-cultural and economic circumstances, fertility desires can be ambiguous, tentative and changeable [46].

In terms of health services, this means that discussions about future fertility could form part of guidelines and counselling protocols of routine HIV/AIDS care. Meeting the SRH needs of PLWHA means more than just counselling those who want to avoid pregnancy; a balanced counselling approach also includes supporting those who desire future childbearing. Health care workers in Kenya rarely give HIV infected women the opportunity to articulate their fertility desires [47]. Proactive screening will ensure that all PLWHA who are considering having children are identified at the earliest opportunity so that appropriate counselling and PMTCT interventions are offered to them. Those who want to limit and space childbearing can then discuss contraception.

Secondly, methods of safer conception could be explored. There are technologies in resource-rich settings that can ensure safer conception for PLWHA such as artificial intrauterine insemination and sperm washing [48]. However, these are out of the reach of resource-poor settings in SSA. The most practical approach at the moment is couple counselling on the ovulation cycle. PLWHA need to be given correct information about the ovulation cycle, when conception is likely to happen and counselled on timed unprotected sexual intercourse during ovulation only and using condoms all the other time, and the associated risks. Although evidence about the feasibility of this method is limited in SSA, a study in Kinshasa, Congo, has shown its effectiveness in preventing HIV transmission to sexual partners [48]. Increasing access to ART in SSA is changing the context of childbearing for PLWHA. ART has been shown to be highly effective in preventing HIV infection among discordant couples, thus presenting a crucial avenue for safer conception among PLWHA [49]. A study that offered timed intercourse combined with ART pre-exposure prophylaxis (PrEP) was able to demonstrate the effectiveness of ART in reducing the transmission risks among HIV negative women and HIV positive men who attempted to conceive [50]. Pronatalist values mean that, for many PLWHA, having children is seen as necessary for a normal and healthy adult life. However, the social rewards of childbearing conflict with moral imperatives of HIV prevention, presenting dilemmas for PLWHA about their future fertility desires and behaviours, which need support from health systems.

## References

[1] UNAIDS (2012) UNAIDS report on the global AIDS epidemic. Geneva: UNAIDS.

[2] WHO (2013) Global update on HIV treatment 2013: results, impact and opportunities: WHO report in partnership with UNICEF and UNAIDS. Geneva: World Health Organization.

[3] Kaida A, Laher F, Strathdee SA, Janssen PA, Money D, et al (2011) Childbearing Intentions of HIV-Positive Women of Reproductive Age in Soweto, South Africa: The Influence of Expanding Access to HAART in an HIV Hyperendemic Setting. Am J Public Health 101: 350–358. 20403884 10.2105/AJPH.2009.177469 PMC3020203

[4] Maier M, Andia I, Emenyonu N, Guzman D, Kaida A, et al (2009) Antiretroviral Therapy is Associated with Increased Fertility Desire, but not Pregnancy or Live Birth, among HIV+ Women in an Early HIV Treatment Program in Rural Uganda. AIDS and Behavior 13: 28–37. 18389364 10.1007/s10461-008-9371-7 PMC3606959

[5] Myer L, Morroni C, Rebe K (2007) Prevalence and Determinants of Fertility Intentions of HIV-Infected Women and Men Receiving Antiretroviral Therapy in South Africa. AIDS Patient Care & STDs 21: 278–285. 17461723 10.1089/apc.2006.0108

[6] Nobrega AA, Oliveira F, Galvao MT, Mota RS, Barbosa RM, et al (2007) Desire for a child among women living with HIV/AIDS in northeast Brazil. AIDS Patient Care & STDs 21: 261–267. 17461721 10.1089/apc.2006.0116

[7] Kanniappan S, Jeyapaul MJ, Kalyanwala S (2008) Desire for motherhood: exploring HIV-positive women’s desires, intentions and decision-making in attaining motherhood. AIDS Care 20: 625–630. 18576164 10.1080/09540120701660361

[8] Oladapo TO, Daniel OJ, Odusoga OL, Ayoola-Sotubo O (2005) Fertility desires and intentions of HIV-positive patients at a suburban specialist center. J Natl Med Assoc 12: 1672–1681. PMC2640756 16396059

[9] Kakaire O, Osinde M, Kaye D (2010) Factors that predict fertility desires for people living with HIV infection at a support and treatment centre in Kabale, Uganda. Reproductive Health 7: 27. 20937095 10.1186/1742-4755-7-27 PMC2964526

[10] Cooper D, Harries J, Myer L, Orner P, Bracken H (2007) “Life is still going on”: Reproductive intentions among HIV-positive women and men in South Africa. Social Science & Medicine 65: 274–283. 17451852 10.1016/j.socscimed.2007.03.019

[11] Cooper D, Moodley J, Zweigenthal V, Bekker L-G, Shah I, et al (2009) Fertility Intentions and Reproductive Health Care Needs of People Living with HIV in Cape Town, South Africa: Implications for Integrating Reproductive Health and HIV Care Services. AIDS and Behavior 13: 38–46. 19343492 10.1007/s10461-009-9550-1

[12] Beyeza-Kashesya J, Kaharuza F, Mirembe F, Neema S, Ekstrom AM, et al (2009) The dilemma of safe sex and having children: challenges facing HIV sero-discordant couples in Uganda. African Health Sciences 9: 2–12. 20842236 PMC2932519

[13] Laher F, Todd C, Stibich M, Phofa R, Behane X, et al (2009) A Qualitative Assessment of Decisions Affecting Contraceptive Utilization and Fertility Intentions among HIV-Positive Women in Soweto, South Africa. AIDS and Behavior 13: 47–54. 19308719 10.1007/s10461-009-9544-z

[14] Smith DJ, Mbakwem BC (2007) Life projects and therapeutic itineraries: marriage, fertility, and antiretroviral therapy in Nigeria. AIDS 21: S37–S41. 10.1097/01.aids.0000298101.56614.af 18090266

[15] Wagner G, Linnemayr S, Kityo C, Mugyenyi P (2011) Factors Associated with Intention to Conceive and its Communication to Providers Among HIV Clients in Uganda. Maternal and Child Health Journal: 1–9. 10.1007/s10995-011-0761-5 21359828

[16] Nattabi B, Li J, Thompson S, Orach C, Earnest J (2009) A Systematic Review of Factors Influencing Fertility Desires and Intentions Among People Living with HIV/AIDS: Implications for Policy and Service Delivery. AIDS and Behavior 13: 949–968. 19330443 10.1007/s10461-009-9537-y

[17] Lam PK, Naar-King S, Wright K (2007) Social support and disclosure as predictors of mental health in HIV-positive youth. AIDS Patient Care & STDs 21: 20–29. 17263655 10.1089/apc.2006.005

[18] UN-HABITAT (2003b) Slums of the World: The Face of Urban Poverty in the New Millenium? Global Urban Observatory. Nairobi: UN-Habitat:.

[19] Zulu E, Beguy D, Ezeh A, Bocquier P, Madise N, et al (2011) Overview of migration, poverty and health dynamics in Nairobi City’s slum settlements. Journal of Urban Health 88: 185–199. 10.1007/s11524-011-9595-0 PMC3132239 21713552

[20] Amuyunzu-Nyamongo M, Okeng’O L, Wagura A, Mwenzwa E (2007) Putting on a brave face: The experiences of women living with HIV and AIDS in informal settlements of Nairobi, Kenya. AIDS Care 19: 25–34. 10.1080/09540120601114618 17364385

[21] Madise NJ, Ziraba AK, Inungu J, Khamadi SA, Ezeh A, et al (2012) Are slum dwellers at heightened risk of HIV infection than other urban residents? Evidence from population-based HIV prevalence surveys in Kenya. Health & Place 18: 1144–1152. 22591621 10.1016/j.healthplace.2012.04.003 PMC3427858

[22] Undie C-C, Ziraba AK, Madise N, Kebaso J, Kimani-Murage E (2009) ‘If you start thinking positively, you won’t miss sex’: narratives of sexual (in)activity among people living with HIV in Nairobi’s informal settlements. Culture, Health & Sexuality 11: 767–782. 10.1080/13691050903105031 19637064

[23] Creswell JW, Plano Clark V (2007) Designing and Conducting mixed methods research. Thousand Oaks, CA: Sage.

[24] Magnani R (1997) Sampling guide. MPACT Food Security and Nutrition Monitoring Project Arlington, Va.

[25] Wekesa E (2012) A new lease of life: sexual and reproductive behaviour of PLWHA the ART era in Nairobi slums. London: London School of economics.

[26] Casterline J, El-Zeini L (2007) The estimation of Unwanted Fertility. Demography 44: 729–745. 18232208 10.1353/dem.2007.0043

[27] Santelli J, Rochat R, Hatfield-Timajchy K, Gilbert BC, Curtis K, et al (2003) The Measurement and Meaning of Unintended Pregnancy. Perspectives on Sexual and Reproductive Health 35: 94–101. 12729139 10.1363/3509403

[28] Gliem JA, Gliem RR (2003) Calculating, Interpreting and Reporting Cronbach’s Alpha Reliability coefficient for Likert-Type scales; 2003 October 8–10, 2003; The Ohio State University, Columbus.

[29] Filmer D, Pritchett LH (2001) Estimating wealth effects without expenditure data –or tears: an application to educational enrollments in states of India. Demography 38: 115–132. 11227840 10.1353/dem.2001.0003

[30] StataCorp (2009) STATA/IC 11.1. College Station, TX, USA.

[31] QSR International (2009) NVivo8. Australia.

[32] Creswell JW (2009) Research Design: Qualitative, Quantitative and Mixed Methods Approaches. Thousand Oaks, California: Sage Publications, Inc.

[33] Howard M (2003) Human Immunodeficiency Virus Infection in Pregnancy. Obstetrics & Gynecology 101: 797–810. 12681888 10.1016/s0029-7844(03)00051-6

[34] KNBS and ICF Macro (2010) Kenya Demographic and Health Surveys (2008). Kenya National Bureau of Statistics [Kenya], Measure DHS, ICF Macro: Calverton, Maryland.

[35] APHRC (2002) Population and health dynamics in Nairobi’s informal settlements. Nairobi: African Population and Health Research Center.

[36] Heys J, Kipp W, Jhangri G, Alibhai A, Rubaale T (2009) Fertility desires and infection with the HIV: results from a survey in rural Uganda. AIDS 23: S37–45. 20081387 10.1097/01.aids.0000363776.76129.fd

[37] Iliyasu Z, Abubakar I, Kabir M, Babashani M, Shuaib F, et al (2009) Correlates of Fertility Intentions Among HIV/AIDS Patients in Northern Nigeria. African Journal of Reproductive Health 13: 71–83. 20690263

[38] Nakayiwa S, Abang B, Packel L, Lifshay J, Purcell D, et al (2006) Desire for Children and Pregnancy Risk Behavior among HIV-Infected Men and Women in Uganda. AIDS and Behavior 10: 95–104. 10.1007/s10461-006-9126-2 16715343

[39] Yeatman S (2009) The Impact of HIV Status and Perceived Status on Fertility Desires in Rural Malawi. AIDS and Behavior 13: 12–19. 19301116 10.1007/s10461-009-9534-1 PMC3856651

[40] Donnell D, Baeten JM, Kiarie J, Thomas KK, Stevens W, et al (2010) Heterosexual HIV-1 transmission after initiation of antiretroviral therapy: a prospective cohort analysis. The Lancet 375: 2092–2098. 10.1016/S0140-6736(10)60705-2 PMC2922041 20537376

[41] Reynolds SJ, Makumbi F, Nakigozi G, Kagaayi J, Gray RH, et al.. (2011) HIV-1 transmission among HIV-1 discordant couples before and after the introduction of antiretroviral therapy. AIDS 25: 473–477 410.1097/QAD.1090b1013e3283437c3283432b. 10.1097/QAD.0b013e3283437c2b PMC3261071 21160416

[42] Cohen MS, Chen YQ, McCauley M, Gamble T, Hosseinipour MC, et al (2011) Prevention of HIV-1 Infection with Early Antiretroviral Therapy. New England Journal of Medicine 365: 493–505. 21767103 10.1056/NEJMoa1105243 PMC3200068

[43] Attia S, Egger M, Müller M, Zwahlen M, Low N (2009) Sexual transmission of HIV according to viral load and antiretroviral therapy: systematic review and meta-analysis. Aids 23: 1397–1404. 19381076 10.1097/QAD.0b013e32832b7dca

[44] WHO (2012) Guidance on couples HIV testing and counselling, including antiretroviral therapy for treatment and prevention in serodiscordant couples: recommendations for a public health approach. Geneva: World Health Organization. 23700649

[45] Mujugira A, Heffron R, Celum C, Mugo N, Nakku-Joloba E, et al.. (2013) Fertility Intentions and Interest in Early Antiretroviral Therapy Among East African HIV-1–Infected Individuals in Serodiscordant Partnerships. JAIDS Journal of Acquired Immune Deficiency Syndromes 63: e33–e35 10.1097/QAI.1090b1013e318288bb318232. 10.1097/QAI.0b013e318288bb32 PMC3624622 23574927

[46] Agadjanian V (2005) Fraught with Ambivalence: Reproductive Intentions and Contraceptive Choices in a Sub-Saharan Fertility Transition. Population Research and Policy Review 24: 617–645.

[47] CRR Fida-Kenya (2008) At risk: Right violations of HIV- positive women in Kenya health facilities. New York: Centre for Reproductive Rights.

[48] Matthews L, Mukherjee J (2009) Strategies for Harm Reduction Among HIV-Affected Couples Who Want to Conceive. AIDS and Behavior 13: 5–11. 19347575 10.1007/s10461-009-9551-0

[49] Mastro T, Cohen M, Rees H (2011) Antiretrovirals for safer conception for HIV-negative women and their HIV-1-infected male partners: how safe and how available?. AIDS 25: 2049–2051. 21997491 10.1097/QAD.0b013e32834c4c62

[50] Vernazza PL, Graf I, Sonnenberg-Schwan U, Geit M, Meurer A (2011) Preexposure prophylaxis and timed intercourse for HIV-discordant couples willing to conceive a child. AIDS 25: 2005–2008 2010.1097/QAD.2000b2013e32834a32836d32830. 10.1097/QAD.0b013e32834a36d0 21716070

